# Swine Backyard Production Systems in Central Chile: Characterizing Farm Structure, Animal Management, and Production Value Chain

**DOI:** 10.3390/ani13122000

**Published:** 2023-06-15

**Authors:** Cecilia Baumberger, Francisca Di Pillo, Pablo Galdames, Cristobal Oyarzun, Victor Marambio, Pedro Jimenez-Bluhm, Christopher Hamilton-West

**Affiliations:** 1 Departamento de Medicina Preventiva, Facultad de Ciencias Veterinarias y Pecuarias, Universidad de Chile, Santa Rosa 11315, Santiago 8820808, Chile; cecilia.baumberger@uchile.cl (C.B.); pgaldames@veterinaria.uchile.cl (P.G.); crisom@ug.uchile.cl (C.O.); victormarambio@veterinaria.uchile.cl (V.M.); 2 Programa de Doctorado en Ciencias Silvoagropecuarias y Veterinarias, Campus Sur Universidad de Chile, Santa Rosa 11315, Santiago 8820808, Chile; 3 Núcleo de Investigaciones Aplicadas en Ciencias Veterinarias y Agronómicas, Facultad de Medicina Veterinaria y Agronomía, Universidad de Las Américas, Sede Providencia, Manuel Montt 948, Santiago 7500972, Chile; fdipillo@udla.cl; 4 Escuela de Medicina Veterinaria, Facultad de Ciencias Biológicas, Facultad de Medicina y Facultad de Agronomía e Ingeniería Forestal, Pontificia Universidad Católica de Chile, Vicuña Mackenna 4860, Santiago 7820436, Chile; pedro.jimenez@uc.cl

**Keywords:** swine, backyard, value chain, Chile

## Abstract

**Simple Summary:**

The central zone of Chile concentrates an important number of backyard production systems, where poultry species are the most commonly present in backyards, followed by swine. There is compelling evidence in the scientific literature indicating the circulation of zoonotic pathogens among backyard poultry and swine. As a result, backyards represent a significant animal-human interface that warrants further investigation. Previous studies have characterized poultry backyards in Chile; however, swine backyards have not yet been characterized. Therefore, the objectives of this study were to characterize swine backyard production systems in the central zone of Chile and to describe the value chain of swine backyard production. We found that backyard production is carried out in the context of the low implementation of biosecurity measures and that in most backyards, there is no animal health management in place; thus, the sanitary status of pigs is usually unknown. Moreover, a significant amount of movement of animals and animal products between different backyards was identified. These results suggest that swine backyards have biosecurity deficiencies that may play an important role in the risk of introduction and dissemination of animal pathogens or the emergence of zoonotic diseases and therefore require special attention.

**Abstract:**

Backyard production systems (BPS) are highly distributed in central Chile. While poultry BPS have been extensively characterized, there remains a notable gap in the characterization of swine BPS in central Chile. In addition, there is evidence that zoonotic pathogens, such as influenza A virus and *Salmonella* spp., are circulating in backyard poultry and pigs. A total of 358 BPS located in central Chile were evaluated between 2013 and 2015 by interviewing farm owners. Severe deficiencies in biosecurity measures were observed. The value chain of swine backyard production identified food, veterinary care (visits and products), and replacement or breeding animals as the primary inputs to the backyard. The most common origin of swine replacements was from outside the BPS (63%). The main outputs of the system were identified as meat and live animals, including piglets and breeding animals. In 16% of BPS, breeding animals were lent to other BPS, indicating the existence of animals and animal product movement in and out of backyard farms. Results from this study indicate that swine BPS in central Chile represents an animal–human interface that demands special attention for implementing targeted preventive measures to prevent the introduction and spread of animal pathogens and the emergence of zoonotic pathogens.

## 1. Introduction

Backyard production systems (BPS) represent the most common form of animal production worldwide [1]. Approximately 13% of the world’s population is linked to small-scale family production systems, which contribute to the household economy and the supply of basic food in rural areas [2]. In Chile, 92% of agricultural farms correspond to small-scale family settings [3]. However, the great volume of Chilean agricultural production is concentrated in the industrial sector. Industrial and backyard production coexist in the central zone of Chile, which includes the Metropolitan, Libertador General Bernardo O’Higgins (LGB O’Higgins), and Valparaiso regions. This area concentrates more than 80% of the total number of pigs in the country, and 2282 swine BPS are located in this same area [4]. Pork production in Chile is primarily concentrated within the industrial sector, characterized by highly integrated large-scale commercial farms that adhere to high biosecurity standards. In contrast, swine BPS in Chile are predominantly small-scale family farms that rear multiple animal species. Among these, poultry species are the most commonly found, followed by swine. However, it is noteworthy that the implementation of biosecurity measures in these BPS is practically absent [5,6]. Backyard production plays a significant role in contributing to the family economy and ensuring food access. Specifically, studies conducted in central Chile have shown that 62% of poultry backyard farmers achieve a positive economic balance from their production activities. Poultry products are primarily intended for household consumption, with some cases also involving trade. Additionally, household poultry consumption increases as BPS distance to markets also increases and is greater for low-income families compared to families of higher per capita income [7].

Although the comprehensive characterization of biosecurity measures in swine BPS is still lacking, the existing literature has extensively documented deficiencies in these practices within poultry BPS. The literature highlights several common deficiencies, including improper handling of mortalities, lack of veterinary treatment for sick animals, and absence of disinfection procedures for people, vehicles, or materials upon entry, among others. In addition, backyard animals may have direct contact with wild birds and neighboring backyard animals [5,6,7,8]. Consequently, the proximity and interaction between people, domestic animals, and wildlife within BPS create conditions that may facilitate the entry of animal pathogens into the backyard environment or contribute to the emergence of zoonotic diseases. This dynamic has the potential to exert a negative impact on both animal and public health [5,9,10,11]. In this context, swine play a pivotal role in the epidemiology of zoonotic infections of influenza A virus (IAV) due to their susceptibility to both avian-origin and mammalian-origin IAV [12,13]. Recent studies have provided evidence of IAV circulation within backyard poultry and swine populations in central Chile. These studies have reported varying prevalence at the farm level, ranging from 27% to 45% across different seasons. Moreover, the detection of positive cases by RT-qPCR has confirmed the presence of IAV in both poultry and swine species [6,8,14]. In addition, an IAV (H1N2) isolated from a pig kept in a BPS located in the Valparaiso region was shown to be a reassortment of human (HA and NA glycoprotein genes) and swine (internal genes) virus genes. This finding presents a significant concern as it signifies a potential risk for individuals in direct contact with animals within these production systems, as well as for public health at large [15]. Additionally, there is evidence of the circulation of foodborne pathogens, such as *Salmonella* spp., in poultry and swine raised in backyards in central Chile, with a reported prevalence of 4.6% at the farm level [16]. Nevertheless, despite the antecedents that highlight the potential role of swine BPS as a point of emergence of zoonotic diseases, these production systems have not yet been deeply characterized in Chile. Consequently, this study aims to achieve the following objectives: (i) to comprehensively characterize swine BPS in the central region of Chile, encompassing aspects of structure, animal management, and implemented biosecurity measures, and (ii) to provide a detailed description of the production value chain.

## 2. Materials and Methods

### 2.1. Study Area and Study Design

**Figure 1:**
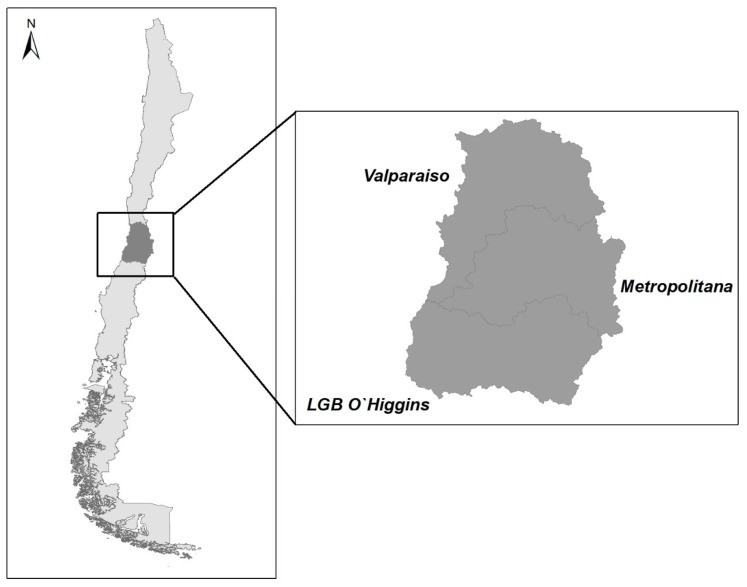
Study area in the central zone of Chile including Metropolitan, Libertador General Bernardo O’Higgins (LGB O’Higgins), and Valparaiso regions.

The target population included BPS-breeding swine located in the central zone of Chile, including the Metropolitan, LGB O’Higgins, and Valparaiso regions (Figure 1). This area of Chile has the highest population of swine and poultry in both industrial and backyard farm settings [4]. A total of 358 BPS were included in the present study. Of these, 71 BPS were selected through a stratified and proportional sampling method that covered all the provinces of the three regions of central Chile, previously described by Bravo-Vasquez and collaborators [8]. The remaining 287 BPS come from a study that aimed to evaluate the risk of introduction of the Porcine Reproductive and Respiratory Syndrome (PRRS) virus, which included all swine BPS in a highly concentrated industrial production area of 100 km^2^ located in Metropolitan and LGB O’Higgins regions.

### 2.2. Farm Data Colection

Farm data were collected using a face-to-face semi-structured interview administered to swine backyard farmers by veterinarians from the Faculty of Veterinary and Livestock Science of the University of Chile between 2013 and 2015. The duration of the interview was approximately 20 min and consisted of open and close questions about BPS structure, handling of the animals, animal movements in and out of the BPS, implemented biosecurity measures, and type and destination of animal products, among others (Table 1). Questionnaire variables considered for the characterization of biosecurity measures implemented in BPS were: (1) presence of functional fences, (2) presence of footbaths, (3) farmer’s handwashing before and after animal handling, (4) presence of poultry or swine in neighboring BPS, (5) proximity to commercial poultry or swine farms, and (6) presence of a water body inside the BPS.

### 2.3. Data Analysis

The variables collected through the interview were presented using descriptive statistics. The comparison of BPS size between different categories was evaluated using non-parametric tests (Kruskal–Wallis) according to the distribution of the response variable. Comparisons of the proportions of categorical variables were made using the Chi-square test. Statistical tests were performed using InfoStat statistical software, and the statistical significance was set at ≤0.05.

To evaluate the relationship between BPS characteristics (main objective of swine breeding, years of swine rearing, and swine management) and biosecurity measures that can be implemented by farmers (functional fences and handwashing before and after animal handling), tests for independence between categorical variables using the Chi-square test were performed.

The questionnaire administered to a total of 71 BPS included variables related to the type, origin, and destination of inputs and outputs of swine production. These data were utilized to construct a conceptual framework aimed at describing the value chain of swine backyard production.

In order to validate the characterization of swine BPS with more recent data, a total of 22 BPS located in Valparaiso (n = 19) and LGB O’Higgins (n = 3) regions that were interviewed between September 2021 and September 2022 were included as a comparison set of data.

## 3. Results

### 3.1. Characterization of Swine BPS: Structure, Animal Handling, and Biosecurity Measures

**Figure 2:**
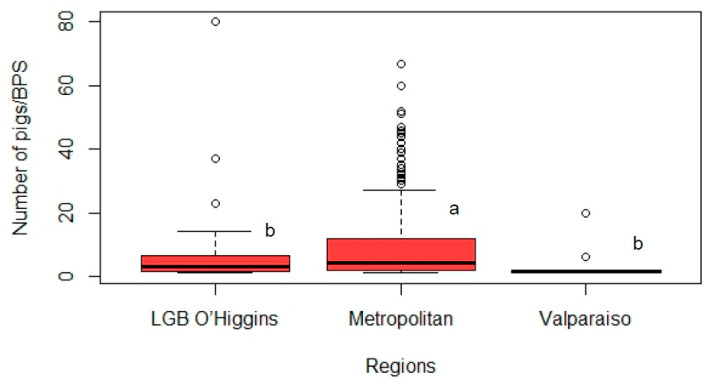
Box plot for swine backyard production system (BPS) size (total number of pigs per BPS) for Libertador General Bernardo O’Higgins (LGB O’Higgins), Metropolitan, and Valparaiso regions in the central zone of Chile ^1^. ^1^ Medians without a common letter differ (*p* < 0.05).

A total of 358 swine backyard farmers from Metropolitan (n = 269), LGB O’Higgins (n = 75), and Valparaiso (n = 14) regions were interviewed. The median BPS size was 4 pigs (minimum = 1; maximum = 80), being greater for Metropolitan (median = 4), compared to LGB O’Higgins and Valparaiso regions, whose medians were 3 and 1.5 pigs per BPS, respectively (*p =* 0.001; Figure 2). Most of the BPS farmers reported household consumption of pig products as the main objective of breeding swine or a mixed objective of household consumption and sale (44% and 46%, respectively). In comparison, only 9% of backyard farmers reported that the objective of pig production was exclusively the sale of animal products, with these proportions being significantly different (*p* < 0.001). Years of swine rearing was equal to or greater than two years for 61% of BPS, while 39% of BPS owners reported having started raising pigs in the last two years. Men were exclusively in charge of pigs in 57% of the BPS, followed by the family (22%) and women (21%). Significant differences were observed in the proportions of various confinement management systems (*p* < 0.001). Among the different types, permanent confinement was found to be the most prevalent (76%), followed by mixed confinement, which allows pigs to roam free for at least part of the day (20%), and free-range systems (4%). The entry and exit of pigs into the farms were commonly reported, with 16% of backyard farmers indicating the practice of lending breeding animals to other backyards. This practice of lending breeding animals to various neighboring backyards was found to be very common. Additionally, the most common origin of swine replacements was from outside the BPS (63%), among which the most common strategy was to acquire animals from neighbors (Table 1).

Regarding animal health management, almost 80% of BPS owners reported not receiving veterinary care. Farmers declared to perform veterinary treatments on pigs in 55% of BPS (mostly antiparasitic drugs); however, less than half of these BPS reported calling a veterinarian when pigs showed clinical signs of disease. Only 18% of backyard farmers reported recognizing clinical signs in pigs, such as lethargy or loss of appetite. Incorrect handling of pig mortalities (any method other than burial or burning, i.e., disposing of the farm or throwing to the garbage) was reported by 20% of BPS (Table 1).

**Figure 3:**
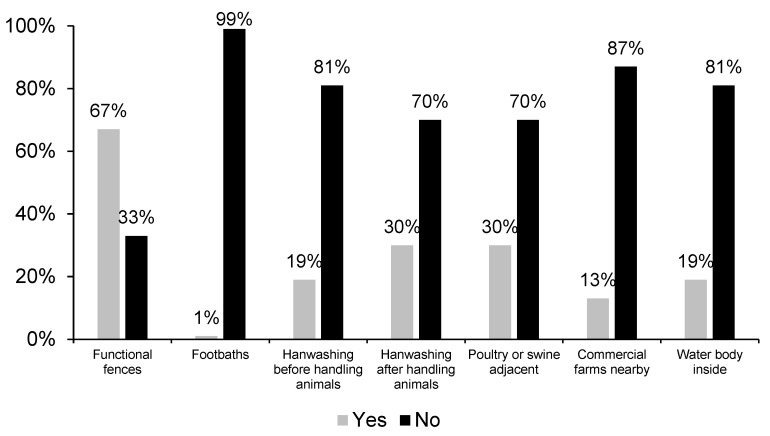
Characterization of biosecurity measures implemented in swine backyard production systems in the central zone of Chile.

In general, the implementation of biosecurity measures was very limited (Figure 3). Almost no BPS footbaths were present at the entrance to the pens, and no handwashing was performed prior to handling the animals in 81% of backyards. Functional fences were absent in 33% of BPS. This practice exhibited a higher frequency in swine backyards with a longer history, in contrast to backyards more recently established and in backyards where more than one family member was in charge of pig management compared to those where men were in charge of animals (*p* < 0.001). Handwashing after handling the animals was reported in 30% of BPS. No association between farm characteristics and the implementation of handwashing before and after animal handling was detected (*p* = 0.363). In addition, BPS size did not differ between backyards that implemented or did not have biosecurity measures (*p* = 0.683; Table 2).

These results are in accordance with recent data (2021–2022) on farm structure, animal management, and implementation of biosecurity measures collected from 22 swine BPS located in central Chile. This fact evidences limited variability in swine backyard farming practices over time. As reported in the previous study period encompassing 358 backyards, the recently interviewed backyards were characterized as small-scale operations, with a median of 3.5 pigs per backyard. The primary objectives of swine breeding in these backyards were also focused on household consumption or a combination of household consumption and commercial sales. Pig management was mainly in charge of men (50%), and permanent confinement was the most frequent pig housing practice reported (77%). Pig movement in or out of the backyard was reported by 27% of farmers, and no veterinary visits were received by most farms (64%; Table S1, Supplementary Materials). Regarding the implementation of biosecurity measures by farmers, none of the 22 farmers interviewed between 2021 and 2022 reported the utilization of footbaths. Moreover, very similar biosecurity deficiencies were identified, including the absence of functional fences in 45% of cases and the lack of handwashing before animal handling in 67% of cases. In 14% of backyards, a water body was present inside the BPS, and most backyards (81%) shared boundaries with neighboring poultry and/or swine backyards. Interestingly, all farmers reported practicing handwashing after animal handling (Figure S1, Supplementary Materials).

### 3.2. Description of Swine BPS Value Chain in Central Chile

**Figure 4:**
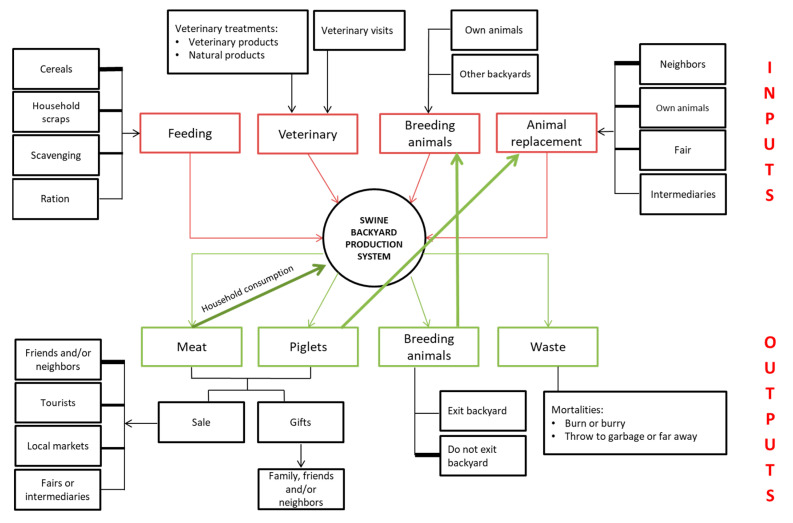
Conceptual framework of type, origin, and destination of inputs and outputs of swine backyard production systems of the central zone of Chile.

The inputs involved in swine backyard production included feed, veterinary visits, veterinary products (including antibiotics, antiparasitics, vitamins, among others), animal replacements (obtained from own sources, other swine BPS, fairs, commercial farms, or intermediaries), as well as breeding animals (acquired from own sources or other swine BPS). Regarding outputs, the main products generated from swine production were live animals (piglets and breeding animals) and meat. The meat was the most valuable product for 55% of swine backyard farmers. Additionally, wastes (dead animals and others) were considered production output. The main buyers of meat and piglets were relatives, neighbors, tourists, local markets, fairs, and intermediaries. Piglets were also used as animal replacements in the same BPS and as gifts to family, neighbors, and friends. Breeding animals were destined for the same BPS or lent to other BPS for reproductive purposes. In the majority of BPS, dead animals were handled correctly (i.e., burning or burial). However, in some BPS, it was reported that dead animals were sold or consumed by household members or thrown away from the BPS (Figure 4).

## 4. Discussion

In the present study, swine BPS in the central zone of Chile were characterized based on general structure, handling of the animals, and implementation of biosecurity measures, together with the description of the value chain of swine backyard production. Results indicate that the implementation of biosecurity measures such as the presence of footbaths and handwashing before and after animal handling are absent in many BPS, along with possible contact of pigs with domestic animals from neighboring backyards and the lack of animal health management. Lack of biosecurity measures has already been reported in backyard poultry rearing in Chile [5,6,7,8,14]; however, swine BPS has not yet been deeply characterized. The results reported in the present study are similar to those reported by other studies carried out in countries where backyard pig production is widely extended due to its significant contribution to rural poverty alleviation and where backyard and small-scale swine farmers have experienced very important economic losses due to the introduction of African swine fever [17,18,19]. Costard and collaborators (2009) described heterogeneity regarding biosecurity practices for small swine farming systems located in different study areas, suggesting that the heterogeneity may be due to differences in culture, climate, and training of farmers [18]. Geographic differences could also be present in different regions of Chile, which throughout its territory includes a wide range of climatic conditions, as well as differences in husbandry practices; therefore, further studies are needed to assess these differences.

As evidenced by recently interviewed backyards used as a comparison dataset (2021–2022) in this study, most practices associated with swine backyard farming show little variability over time. Swine backyards continue to be a small farm setting with important biosecurity deficiencies. The absence of structure or biosecurity improvements over time was also observed for poultry backyard farms in central Chile, where deficiencies in the implementation of biosecurity measures are constant results reported by studies from about a decade ago [5,6,7,8]. Interestingly, the implementation of handwashing after animal handling showed a significant increase when comparing the 2013–2015 dataset (30% of farmers reported this practice) to recently interviewed farmers during 2021–2022 (100% of farmers reported this practice). However, handwashing before animal handling remains an infrequent practice (33%). Considering that the period of administration of interviews with farmers for the more recent set of data was more than one year after the onset of the COVID-19 pandemic, the preventive hygiene measures recommended to decrease the risk of virus transmission may explain these results.

Regarding animal confinement, different management is present depending on the species bred. Studies conducted on poultry BPS in Chile have reported that approximately only 10% of these BPS implement permanent confinement for poultry [5,7]. On the contrary, the present study found that permanent confinement is the most common practice in swine BPS. However, due to the type of pens found in swine backyard production (most are not roofed), contact between domestic and wild animals is still important.

The production value chain presented in this study reveals a significant flow of animals into and out of backyard farms. It was found that the predominant source of animal replacements was external to the farm, indicating a substantial movement of animals. The sanitary status of backyard pigs is usually unknown due to the lack of veterinary care and animal health management, representing a risk for disseminating animal diseases. Most backyard farmers reported that the main objective of swine backyard production is household consumption of products. Meat is the most important output of swine production, and slaughter is carried out in the backyard without the inspection of meat products by veterinary doctors, which represents a risk for people’s exposure to foodborne pathogens, such as *Salmonella* spp. [16].

The risk of introduction and dissemination of animal pathogens in swine BPS evidence the challenges of disease control in backyards [10,19,20]. In the present study, a significant proportion (one-third) of backyard farmers reported the absence of functional fences, suggesting that backyard swine may come into contact with domestic or wild animals from outside the BPS, thus highlighting a context of inadequate biosecurity measures. This holds significant importance, especially after the confirmation of outbreaks of African swine fever in BPS within two distinct geographic regions of the Dominican Republic in July 2021. These outbreaks mark the first occurrence of African swine fever in the Americas since 1980, underscoring the need for heightened surveillance and preventive measures in the region [21,22,23]. Given the high risk of animal disease dissemination in swine BPS, backyards pose a distinct challenge in disease eradication programs. This is exemplified by the epidemiological strategy that played a pivotal role in declaring a significant portion of Colombian territory free from Classical swine fever, wherein effective backyard management played a fundamental role [24]. In addition, swine production farms have a potential role in the emergence and spread of zoonotic pathogens with very adverse impacts on public health. This was the case of the H1N1 (pdmH1N1) influenza pandemic that emerged in Mexico in 2009, where a new viral variant was initially transmitted from pigs to people and later between people through the respiratory route, giving rise to the first pandemic of the 21st century [12,25,26]. In this regard, previous findings of IAV circulation in pigs kept in BPS in central Chile further highlight the importance of our results [6,14,15]. The detection of a reassortant human–swine IAV H1N2 in a backyard pig within the central zone of Chile, exhibiting in vivo and in vitro replicative capability and droplet transmission in a ferret model, highlights the potential risk of exposure to animal-origin IAV for backyard farmers and household members [15]. The limited adoption of biosecurity measures in the management of backyard swine, coupled with the significant inter-farm animal movement observed in the current study, along with previous evidence of IAV circulation in backyard animals, underscores the potential role of swine backyards in the emergence of zoonotic diseases. These findings highlight the need for further attention and preventive measures in these farm settings. Results from this study might contribute as an important input for the development of guidelines on management and biosecurity practices in swine BPS, as well as for educational activities of backyard farmers in disease recognition and animal health management.

## 5. Conclusions

The movement of animals and animal products within and outside swine BPS in Central Chile, coupled with the inadequate implementation of biosecurity measures, highlights a substantial risk of introducing and disseminating animal diseases, including the emergence of zoonotic pathogens. As a result, swine BPS in the central zone of Chile should be included in targeted surveillance programs and preventive measures to mitigate the potential negative impact on public health associated with the emergence of zoonotic pathogens.

## Figures and Tables

**Table 1:** Questionnaire variables used for the characterization of swine backyard production systems in the central zone of Chile.

Variable	Categories	Number	Percentage
Main objective of swine breeding	Household consumption	157	44%
	Sale	31	9%
	Household consumption and sale	163	46%
	No product obtained	3	1%
	Total	354	
Years of swine rearing	Less than 2 years	133	39%
	Between 2 and 10 years	98	28%
	More than 10 years	113	33%
	Total	344	
Confinement	Free range	15	4%
	Mixed	69	20%
	Permanent	268	76%
	Total	352	
Swine management	Woman in charge	74	21%
	Man in charge	200	57%
	Family in charge	75	22%
	Total	349	
Veterinary care	No veterinary care	272	78%
	Veterinary care at least once a year	76	22%
	Total	348	
Feeding	Grains	58	17%
	Swine feed	15	5%
	Scavenging and household scrap	21	6%
	Mixed	241	72%
	Total	335	
Water	Potable sources	303	87%
	Environmental sources	47	13%
	Total	350	
Mortalities handling	Bury	208	63%
	Burn	28	9%
	Throw into the garbage	14	4%
	Throw far away	17	5%
	Household consumption or sale	4	1%
	Nothing	8	3%
	Mixed	24	7%
	No mortalities reported	25	8%
	Total	328	
Movement of swine in or out of the BPS	Yes	39	16%
	No	202	84%
	Total	241	
Replacement	Own offspring	123	37%
	Neighbors	146	43%
	Own offspring and neighbors	32	10%
	Markets or other	21	6%
	Mixed	15	4%
	Total	337	

**Table 2:** Relationship between swine backyard production systems (BPS) characteristics and implementation of biosecurity measures.

BPS Characterization Variables	Functional Fences		*p*-Value	Handwashing before and after Animal Handling		*p*-Value
	Yes; n (%)	No; n (%)		Yes; n (%)	No; n (%)	
Main objective of swine breeding			0.106			0.593
Household consumption	109 (46%)	48 (43%)		24 (40%)	133 (46%)	
Sale	25 (10%)	5 (4%)		7 (12%)	23 (8%)	
Household consumption and sale	104 (44%)	58 (51%)		29 (48%)	132 (45%)	
No product obtained	1 (0%)	2 (2%)		0 (0%)	3 (1%)	
Total	239	113		60	291	
Years of swine rearing			<0.001			0.557
Less than 2 years	108 (46%)	23 (22%)		26 (44%)	105 (37%)	
Between 2 and 10 years	69 (29%)	29 (27%)		14 (24%)	83 (30%)	
More than 10 years	59 (25%)	54 (51%)		19 (32%)	94 (33%)	
Total	236	106		59	282	
Swine management			<0.001			0.363
Man in charge	155 (65%)	44 (40%)		34 (58%)	165 (57%)	
Woman in charge	49 (21%)	24 (22%)		9 (15%)	63 (22%)	
Family in charge	34 (14%)	41 (38%)		16 (27%)	59 (21%)	
Total	238	109		59	287	
Pigs/BPS, median (range)	3.5 (1; 67)	4.0 (1; 80)	0.683	3.0 (1; 60)	4.0 (1; 80)	0.966

## Data Availability

The data presented in this study are available on request from the corresponding author.

## Supplementary Materials

The following supporting information can be downloaded at: https://www.mdpi.com/article/10.3390/ani13122000/s1, Figure S1: Characterization of biosecurity measures implemented in swine backyard production systems in the central zone of Chile, 2021–2022 (n = 22); Table S1: Characterization of swine backyard production systems in the central zone of Chile, 2021–2022 (n = 22).
