# Supplementary material for: Swine Backyard Production Systems in Central Chile: Characterizing Farm Structure, Animal Management, and Production Value Chain
Source: Animals (Basel). 2023 Jun 15;13(12):2000. doi: 10.3390/ani13122000 (PMC10295241; doi:10.3390/ani13122000)

Supplemental material

Table S1. Characterization of swine backyard production systems in the central zone of Chile, 2021 - 2022 (n = 22).

Variable	Categories	Number	Percentage
Main objective of swine breeding	Household consumption	9	41%
	Sale	2	9%
	Household consumption and sale	8	36%
	No product obtained	3	14%
	Total	22	
Years of swine rearing	Less than 2 years	5	23%
	Between 2 and 10 years	6	27%
	More than 10 years	11	50%
	Total	22	
Confinement	Free range	0	0%
	Mixed	5	23%
	Permanent	17	77%
	Total	22	
Swine management	Woman in charge	8	36%
	Man in charge	11	50%
	Family in charge	3	14%
	Total	22	
Veterinary care	No veterinary care	14	64%
	Veterinary care at least once a year	8	36%
	Total	22	
Feeding	Grains	3	13%
	Swine feed	1	5%
	Scavenging and household scrap	1	5%
	Mixed	17	77%
	Total	22	
Water	Potable sources	22	100%
	Environmental sources	0	0%
	Total	22	
Mortalities handling	Bury	4	18%
	Burn	0	0%
	Throw to the garbage	0	0%
	Throw far away	1	5%

	Household consumption or sale	0	0%
	Nothing	0	0%
	Mixed	0	0%
	No mortalities reported	17	77%
	Total	22	
Movement of swine in or out the BPS	Yes	6	27%
	No	16	73%
	Total	22	
Replacement	Own offspring	12	54%
	Neighbors	5	23%
	Own offspring and neighbors	4	18%
	Markets or other	1	5%
	Total	22	

Figure S1. Characterization of biosecurity measures implemented in swine backyard production systems in the central zone of Chile, 2021 – 2022 (n = 22).

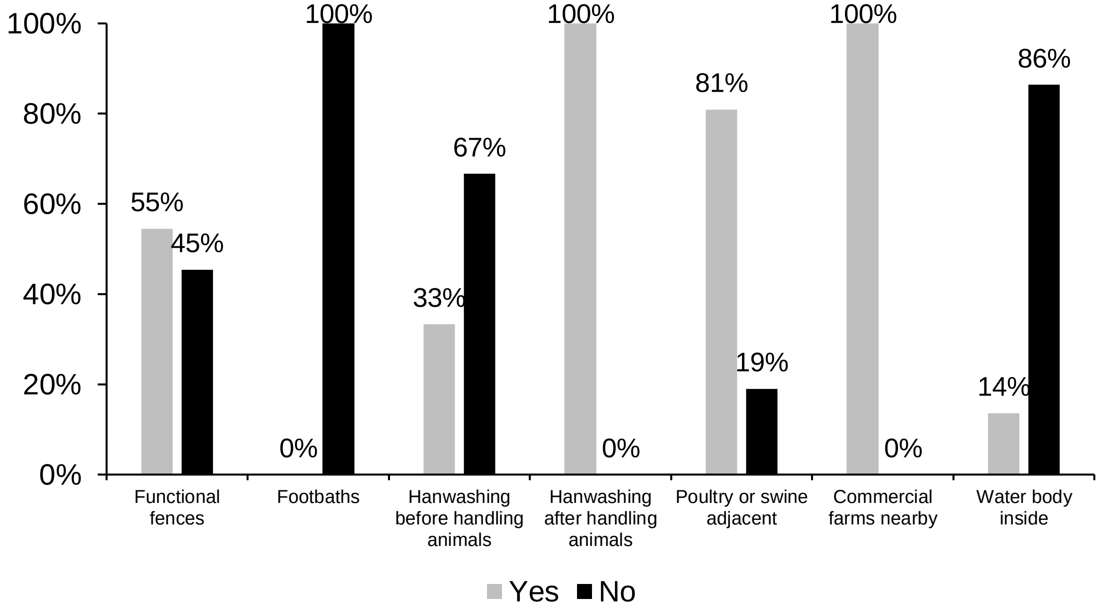

Supplement: Supplementary file 1 [file animals-13-02000-s001.zip › animals-2368582-supplementary.pdf]
